# Internal-Modified Dithiol DNA–Directed Au Nanoassemblies: Geometrically Controlled Self–Assembly and Quantitative Surface–Enhanced Raman Scattering Properties

**DOI:** 10.1038/srep16715

**Published:** 2015-11-19

**Authors:** Yuan Yan, Hangyong Shan, Min Li, Shu Chen, Jianyu Liu, Yanfang Cheng, Cui Ye, Zhilin Yang, Xuandi Lai, Jianqiang Hu

**Affiliations:** 1 Department of Chemistry, College of Chemistry and Chemical Engineering, South China University of Technology, Guangzhou, 510640, China; 2 Department of Physics, Xiamen University, Xiamen 361005, China

## Abstract

In this work, a hierarchical DNA–directed self–assembly strategy to construct structure–controlled Au nanoassemblies (NAs) has been demonstrated by conjugating Au nanoparticles (NPs) with internal–modified dithiol single-strand DNA (ssDNA) (Au–B–A or A–B–Au–B–A). It is found that the dithiol–ssDNA–modified Au NPs and molecule quantity of thiol–modified ssDNA grafted to Au NPs play critical roles in the assembly of geometrically controlled Au NAs. Through matching Au–DNA self–assembly units, geometrical structures of the Au NAs can be tailored from one–dimensional (1D) to quasi–2D and 2D. Au–B–A conjugates readily give 1D and quasi–2D Au NAs while 2D Au NAs can be formed by A–B–Au–B–A building blocks. Surface-enhanced Raman scattering (SERS) measurements and 3D finite–difference time domain (3D-FDTD) calculation results indicate that the geometrically controllable Au NAs have regular and linearly “hot spots”–number–depended SERS properties. For a certain number of NPs, the number of “hot spots” and accordingly enhancement factor of Au NAs can be quantitatively evaluated, which open a new avenue for quantitative analysis based on SERS technique.

DNA–based programmable assemblies of metal nanoparticles (NPs) are an effective avenue to construct regular and desirable nanoarrays, which offer tailored and enhanced optical and electrical properties and have great potential in molecular recognition, surface enhanced Raman spectroscopy (SERS), plasmon–enhanced fluorescence, nonlinear optic, nanocircuitry, biomolecular electronics, nanobiotechnology and nanomedicine and solar cell^1^,^2^,^3^,^4^,^5^. The nanoassemblies (NAs) properties are known to be closely dependent on its interparticle spacing, size and shape^6^,^7^. For example, it has theoretically been demonstrated that the SERS signals induced by metal NAs will sharply increase when the interparticle spacing (generally less than 3.5 nm) of metal NAs decreases^8^. Besides, it is also important to design metal NAs with specific geometrical structures or shapes for improving NAs performance. Currently, one–dimensional (1D) metal NAs have been easily prepared by the existing DNA–directed strategies^9^. For example, dimer and trimer nanostructures have assembled successfully through enzymatic ligation to link the NPs coupled with single–strand DNA (ssDNA)^10^. The interparticle distance and ensemble SERS properties of Au nanodimers have been successfully tailored through varying the molecule length of DNA bridge^11^. By varying the amount of DNA–binding peptide attached to Au NPs and using specifically designed oligonucleotides, Coomber *et al.* successfully obtained 1D Au NAs with controlled lengths and demonstrated that the 1D NAs possess adjustable surface plasmon resonance (SPR) properties^12^. Moreover, micrometer–long 1D nanoarrays can be also assembled up to 4 μm by long (more than 10000 bases), linear and tandemly repetitive single DNA strands^7^.

DNA molecules can assemble itself not only into a linear template but also easily into 2D geometrical structure due to its well–defined geometrical structure, high predictability and programmable intra/intermolecular interactions, which have potential in 2D metal NPs assembly^13^,^14^. Yan’s group effectively constructed 2D Au with periodic and controllable interparticle spacing by coupling ssDNA–functionalized Au NPs to DNA scaffolds^15^. Kiehl’s group also obtained 2D Au NAs through using 2D DNA scaffolds preassembled on a surface^16^. The preassembled 2D DNA strategy can effectively “fix” interparticle distance and assembly shape. Besides, through linking other materials to Au NPs surface, such as silica–gel and binary mixture of 11–mercaptoundecanoic and 4–mercaptophenylacetic acid, ssDNA–Au NPs can also assemble into 2D Au nanoarrays^9^,^17^,^18^. Recently, Gang’s group has also successfully assembled DNA–coated 2D Au nanoarrays at liquid interfaces by controlling the interaction between positively charged lipid layer and negatively charged DNA shells of particles^19^. However, the DNA-directed self-assembly strategy to simultaneously produce 1D, quasi–2D (between 1D and 2D) and 2D Au NAs is still rare to date although there are many methods for self-assembling 1D or quasi–2D or 2D Au NAs. Moreover, the existing strategies to simultaneously produce 1D, quasi–2D and 2D Au NAs are much complex, time-consuming, low-yield and only available to assemble a small quantity of Au NPs^13^. Therefore, it is very urgent to design a simple, effective and general DNA-directed self-assembly strategy for simultaneously constructing 1D, quasi–2D and 2D Au NAs.

SERS is a powerful spectroscopy technique that can provide non–destructive and ultra–sensitive characterization down to single molecular level and significant applications in examination of food safety, drugs, explosives and environment pollutants^20^. The SERS sensitivity highly depends on not only the composition, size, shape and environment medium of metal NPs but also its aggregate size, shape and interparticle spacing^21^,^22^,^23^,^24^,^25^. It is well known that almost all effective SERS–active systems are derived from aggregating NPs to date^26^,^27^. This is because in aggregated noble metal nanostructures, surface plasmon oscillations on proximal particles can couple one another via near–field coupling effect and thus inducing extraordinarily strong Raman signals^28^,^29^,^30^. Actually, SERS is not a single particle’s behavior and the near–field coupling is required to obtain huge electromagnetic (EM) enhancement^31^. The coupling is easily formed in the junctions (i.e., “hot spots”) of metal NP aggregates (interparticle distance is generally less than 3.5 nm)^32^. Dimers or trimers are the simplest NP aggregations, which are, nonetheless, not ideal SERS substrates due to their poor stability, reproducibility and controllability. Firstly, it is still difficult to accurately fix the distance between the neighboring NPs for the aggregating dimers or trimers through random aggregation of Au NPs in substrates or solutions although it have been achieved by DNA-directed Au self-assembly strategy^18^. Besides, for an isolated dimer or trimer, the enhancement factor is highly dependent on the light–polarization. A small difference of the polarization direction will result in very different SERS signal.

For closely packed metal NPs (more than three NPs aggregates), although possessing lots of “hot spots” and thus inducing huge EM enhancement that is possibly independent on the light–polarization^33^, it is still difficult to evaluate the inducing SERS enhancement due to their uncertain structure (e.g., interparticle distance and aggregate size and shape). Moreover, the close packing of metal NPs can lead to damping of the SERS signal due to the electron exchange among NPs. DNA-directed self-assembly is an effective avenue to construct closely packed metal NPs with well-defined interparticle space, which can induce relatively stable and controlled SERS signal^34^,^35^,^36^. However, to further broaden SERS applications, it would be ideal to produce modest, reproducible and stable SERS substrates with quantitative enhancement. Herein, we design a simple, effective and general DNA–directed self–assembly strategy to construct structure–controlled Au NAs with well–defined interparticle distance. In this self–assembly, Au NPs coupled with internal–modified dithiol ssDNA (B–A) and its complementary terminal–modified thiol ssDNA (A´ and B´) are served as assembly units. It is found that the B–A–based Au NPs conjugates and molecule quantity of thiol–modified ssDNA grafted to Au NPs play critical roles in the assembly of geometrically controlled Au NAs. Through carefully selecting the self–assembly units, 1D, quasi–2D and 2D Au NAs can be obtained. The geometrically controllable Au NAs have regular SERS properties that are linearly dependent on their “hot spots” numbers, which has been confirmed by the SERS measurements and theoretical calculation.

## Results and Discussion

### Geometrically controlled self–assembly of Au NPs

**Figure 1:**
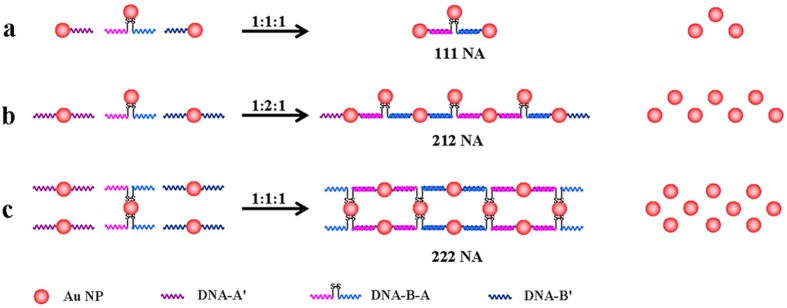
Schematic illustration of the formation of (**a**) 111, (**b**) 212 and (**c**) 222 Au NAs, respectively. Wherein, 1 and 2 mean ssDNA strand number conjugated to Au NP. The concentration ratios of three Au–DNA conjugates are (**a**,**c**) 1:1:1 and (**b**) 1:2:1 for assembling Au NAs, respectively.

In our strategy, it is very important to employ internal–modified dithiol ssDNA for producing geometrically controlled Au NAs due to its assembly flexibility and diversity^37^. Different hierarchical self–assembly strategies for geometrically controlled Au NAs from 1D to 2D are illustrated in Fig. 1. The internal–modified dithiol B–A has two equal–length arms (36 bp/36 bp, Fig. S1), two ends of which can hybridize with 5´ and 3´ end thiol–modified A´ (36 bp) and B´ (36 bp), respectively. Spherical Au NPs are functionalized respectively with A´, B´ and A–B (denoted as Au–A’, Au–B’ and Au–A–B conjugates, respectively), which are used as assembling units of Au NAs. For large–sized Au NPs (≥10 nm), it is usually required to link additional long ssDNA strands for extracting Au–DNA conjugates with well–defined numbers of ssDNA by electrophoresis separation^38^.

Six EXT–DNA strands (each for 79 bp) are chosen to lengthen three thiol–modified ssDNA

strands. For instance, the B–A is first lengthened with EXT–B(1), B(2), B(3), B(4), B(5) and B(6) (Table S1). Then the as–prepared lengthened DNA strands are conjugated with Au NPs, and the resulting Au–DNA conjugates are separated by agarose gel electrophoresis (AGE). Finally, the underlying Au–DNA conjugates can be utilized to assemble into geometrically controlled Au NAs only through selecting assembling units and concentration ratios. For example, 1D 111 Au NAs are obtained through mixing Au–A´, Au–B´ and Au–B–A conjugates with 1:1:1 concentration ratio (Fig. 1a). Also, 1:2:1 of A´–Au–A´, B´–Au–B´ and Au–B–A conjugates is used to construct quasi–2D 212 Au NAs (Fig. 1b). Further, using A–B–Au–B–A conjugates with two medium–dithiol–modified ssDNA strands, well–ordered and periodic 2D Au NAs can be formed (Fig. 1c).

### Gel electrophoresis of Au–DNA conjugates

**Figure 2:**
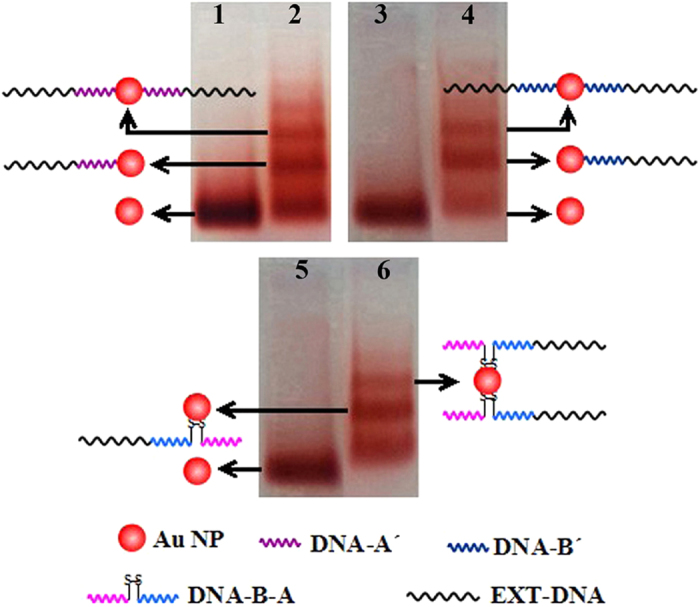
Agarose gel purifications of the Au NPs conjugated with three thiol–modified DNA strands, respectively. Lanes 1, 3 and 5 are pure Au NPs; Lanes 2, 4 and 6 correspond to Au–A´–EXT–DNA, Au–B´–EXT–DNA and Au–B–A–EXT–DNA, respectively.

Gel electrophoresis separation is well known to be the key step for generating high–quality DNA–directed Au NAs. Figure 2 illustrates agarose gel purification of the Au NPs conjugated with three kinds of thiol–modified DNA strands. Lanes 1, 3 and 5 correspond to reference samples of the BSPP–capping Au NPs with negative charges. Three or more bands can be distinctly discerned in Lanes 2, 4 and 6, indicating the successful separation of different Au–DNA conjugates. The second and third bands show electrophoresis purification of three Au–DNA conjugates, corresponding Au–A´ and A´–Au–A´, Au–B´ and B´–Au–B´ or Au–B–A and A–B–Au–B–A, respectively. Of special emphasis here, the successful electrophoresis separation of the Au–DNA conjugates prepared by internal–modified dithiol ssDNA strands (Fig. 2, Line 6) is an important breakthrough in comparison with the previous work that could only obtain an Au–DNA conjugate mixture^12^. This may be because that the –S–S– of B–A connected with alkane chains (Fig. S1) have lower steric hindrance than that coupled with cycloalkane when Au NPs approach to internal dithiol (–S–S–) of the DNA. Carefully cutting and extracting the second and third bands in Lane 2 of Fig. 2, Au–A´ and A´–Au–A´ conjugates containing lengthened DNA strands could be obtained. The lengthened DNA strands could be removed through heating to 55 °C, thus pure and well–defined Au–A´ and A´–Au–A´ conjugates could be acquired, respectively. Similarly, Au–B´, B´–Au–B´, Au–B–A and A–B–Au–B–A conjugates could be also extracted through the same procedures.

### Transmission electron microscopy (TEM) images and ultraviolet-visible (UV–vis) spectra of geometrically controlled Au NAs

**Figure 3:**
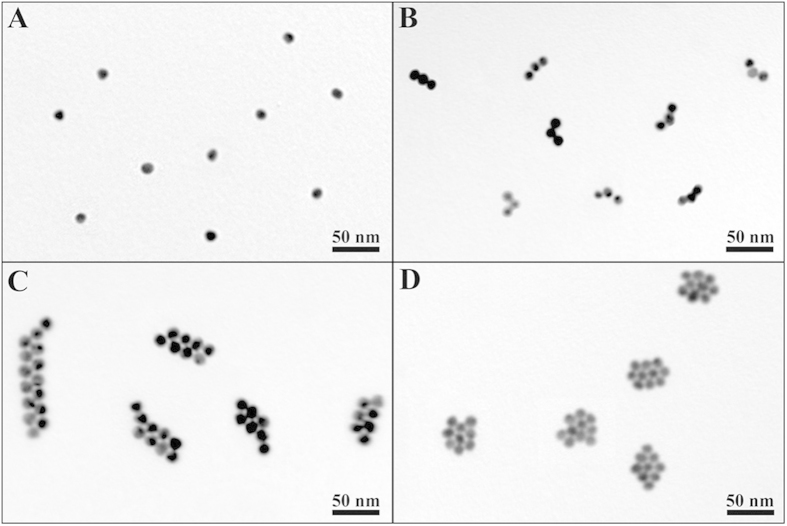
TEM images of (**A**) Au NPs and (**B–D**) 111, 212 and 222 NAs constructed by the present methods.

Using internal–modified dithiol ssDNA–Au NP conjugates as self–assembly units, geometrically controlled Au NAs can be constructed through its two arms (Fig. 3). Figure 3A gives typical TEM image of Au NPs prepared by the present method. The Au NPs have relatively uniform size of 14.4 ± 1.1 nm. To verify component and size distribution of NPs, X-ray photoelectron spectroscopy (XPS) and dynamic light scattering (DLS) were performed. The results indicate that the NPs are Au and have well monodispersed (Figs S2,S3). When Au–B–A conjugate hybridize with Au–A´ and Au–B´ conjugates by 1:1:1 concentration ratio, the 111 Au NAs can be successfully acquired (Fig. 3B). Coupling A´–Au–A´, Au–B–A and B´–Au–B´ conjugates (1:2:1 concentration ratio), Au NAs with two parallel rows can be obtained (Fig. 3C). The 212 NAs had zigzag shape and quasi–2D structure, with length up to more than 300 nm. If Au–B´, B–A–Au–B–A and Au–A´ conjugates are used and their concentration ratio is 2:1:2, it is predicted that 121 Au NAs with tetrahedral structure should be obtained in aqueous solution due to its most stable structure although 2D trapezoidal shape is seen in the TEM image of 121 Au NAs (Fig. S4). Very interestingly, using A´–Au–A´ and B´–Au–B´ to hybridize A–B–Au–B–A (1:1:1 concentration ratio), 2D Au NAs with three parallel rows are obtained (Fig. 3D). Without further purification, these Au NAs all have relatively high yield (more than 74%) and well monodispersed (Fig. S3). It is important to note that the conjugate concentration ratio plays an important role in the yield of Au NAs. The conjugate concentration ratios designed here justly make three DNA-Au conjugates match. If other concentration ratios are used, the yield of Au NAs will greatly lower (Fig. S5).

Geometrical structures of Au NAs can be also well characterized by UV–vis spectroscopy, as shown in Fig. S6. The Au NPs give a UV-vis absorption peak at about 522 nm (black line, Fig. S6). When the Au NPs assemble into 111 NAs, its UV-vis absorption peaks slightly red-shift from *ca.* 522 to 528 nm (red line, Fig. S6). Furthermore, the UV-vis absorption peaks of the 212 and 222 NAs red-shift to longer wavelength at approximately 530 and 532 nm (green and blue lines, Fig. S6), respectively. These indicate that the UV-vis absorption properties of the Au NAs are dependent on their assembly structures. Along with varying assembly structures from 1D to quasi-2D and 2D, the UV-vis absorption peaks of the Au NAs have slightly red-shift. The slight red shift is possibly caused by the interparticle coupling that is associated with NP number and interparticle gap (nearly same) in the Au NAs^39^. However, the red-shift of the UV-vis absorption peaks has not as much as expected, which is perhaps due to their quasi-3D structures in solution.

### SERS properties of geometrically controlled Au Nas

**Figure 4:**
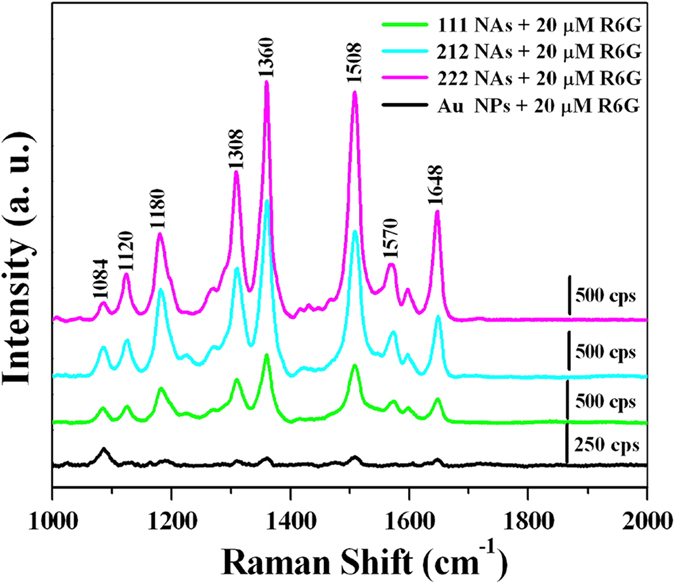
SERS spectra of 20 μM R6G, as the probe molecules, adsorbed on the Au NPs (black line), 111 NAs (green line), 212 NAs (blue line) and 222 NAs (purple line), respectively.

**Figure 5:**
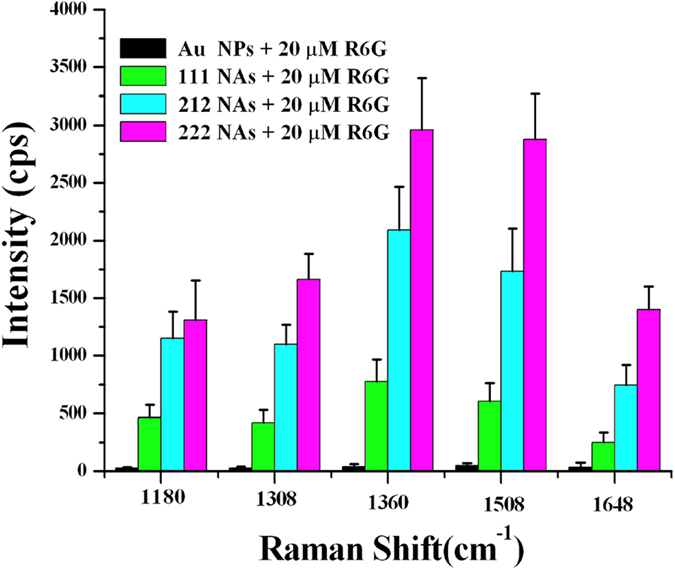
Intensity comparison of SERS signals at 1180, 1308, 1360, 1509 and 1648 cm^−1^ of 20 μM R6G adsorbed on Au NPs and 111, 212 and 222 NAs, respectively.

Self–assembled metal NPs can induce giant surface electric field enhancement by localized surface plasmon coupling at particle–particle junctions, which is very attractive for SERS applications^27^,^40^. Figure 4 shows SERS spectra of 20 μM R6G, the probe molecules adsorbed on the Au NPs and NAs. Eight Raman peaks of ca. 1084, 1120, 1180, 1308, 1360, 1508, 1570 and 1648 cm^−1^ can be distinctly discerned in Fig. 4, and are intrinsic to R6G^27^. Raman signal observed at approximately 1180 cm^–1^

results from C–C stretching vibrations and these bands at around 1308, 1360, 1508, 1570 and 1648 cm^−1^ can be ascribed to the aromatic C–C stretching vibrations^41^. Additionally, the band at 1084 cm^−1^ can be assigned to the residual coated molecules^42^. These Raman signals were acquired through averaging 5 Raman spectra measured at 5 different spots. It can be inferred from Fig. 4 that the SERS signals of R6G on the Au NAs are much stronger than those on the Au NPs. Moreover, the SERS intensities of R6G on the Au NAs gradually increase as its geometrical structure changes from 1D to quasi–2D and 2D. Figure 5 compares the intensities of the SERS signals at about 1180, 1308, 1360, 1508 and 1648 cm^−1^ of 20 μM R6G adsorbed on Au NPs and 111, 212 and 222 NAs. The relative enhancement factors (REF) between the Au NAs and Au NPs can be calculated by contrasting the SERS intensities of the band at 1360 cm^−1^. And the REF of the 111, 212 and 222 Au NAs are about 18, 49 and 69, respectively.

### SERS theoretical calculation of geometrically controlled Au NAs

**Figure 6:**
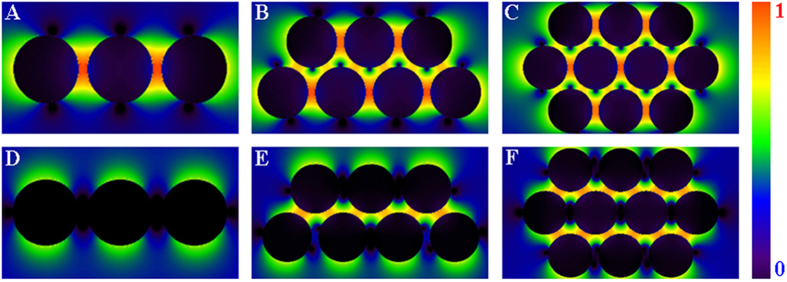
3D–FDTD simulated electric field patterns of (**A–C**) X–polarization and (**D–F**) Y–polarization of the 111, 212 and 222 NAs, respectively. (**A**,**D**) 111 NAs, (**B**,**E**) 212 NAs and (**C**,**F**) 222 NAs. The electric field patterns have been normalized, in which 1 and 0 represent the strongest and the weakest electric field enhancement.

To understand the SERS behavior of the Au NAs, three–dimensional finite–difference time domain (3D–FDTD) method is used to calculate the electromagnetic field by numerically solving Maxwell’s equations^43^. In this simulation, a 632.8 nm laser beam is vertical to the plane of the Au NAs, with its polarization along the X– and Y–axis, respectively. The original electrical amplitude of the exploited plane wave is 1 V/m in the simulation, and the optical constant of Au is taken from a reference^44^. The 3D–FDTD simulated electric field patterns of X– and Y–polarization of 111, 212 and 222 NAs are illustrated in Fig. 6. Obviously, the electromagnetic field whose strength depends on both light polarization and geometrical structure of the NAs is particularly strong in the “hot spots” between Au NPs. It can be clearly seen from Fig. 6 that there are obvious difference between X– and Y–polarization. For the X–polarization, the electromagnetic field is mainly concentrated on horizontal junctions among Au NPs. Accordingly, the hot spots in the Y–polarization arise from the junctions among vertical particles.

The SERS enhancement factors (EFs) of the 111, 212 and 222 NAs were calculated by 3D-FDTD (Table S2). According to the SERS theory^45^, the SERS EFs are roughly proportional to |E_loc_/E_0_|^4^, where E_loc_ and E_0_ are the amplitudes of the localized electric field and incident electric field, respectively. The difference between X– and Y–polarization is strongly dependent on geometrical structure of the NAs (Fig. 6 and Table S2). For instance, the X–polarization value (17.4 V/m) of the 1D 111 NAs is about 3 times larger than that (5.2 V/m) of its Y–polarization. Varying the NAs geometrical structure from 1D to quasi 2D, its Y–polarization value (17.5 V/m) starts to approach to its X–polarization (10.2 V/m). Furthermore, the 2D 222 NAs with three–parallel structure have similar X– and Y–polarization values (14.1 and 12.3 V/m, respectively). The corresponding theoretical EFs of 111, 212 and 222 NAs are 9.2 × 10^4^, 9.4 × 10^4^ and 4.0 × 10^4^ for X–polarized incident light and 7.3 × 10^2^, 1.1 × 10^4^ and 2.3 × 10^4^ for the Y–polarized, respectively. It can be clearly seen from Table S2 that the EFs of the three Au NAs are all approximately four orders of magnitude except for 111 NAs of Y-polarization. Therefore, due to the similar interparticle distance (approximately 2.0 nm) that plays an essential role in coupling SPR intensity, the EFs induced by the Au NAs are mainly determined by their “hot spots” numbers.

### Statistic model of “hot spots” in Au NAs

X– and Y–polarization lights are used to simulate natural light (unpolarized) in our system. The total “hot spots” number of Au NAs under natural light can be calculated through simply summing the number of “hot spots”, which corresponds the reddest area in the electromagnetic field distribution along its X– and Y–polarization directions. To explain enhancement of SERS signals of the Au NAs, a statistic model is proposed. Provided that 3N Au NPs are employed to assemble the Au NAs, different assembly modes will generate structures with different “hot spots” numbers. The monodispersed Au NPs have a large interparticle distance (Fig. 3A), thus its inducing SERS signal is very weak (Fig. S7). Therefore, the “hot spots” number of the 3N monodispersed Au NPs can be taken as zero.

If the 3N Au NPs are utilized to assemble into 111, 212 and 222 Au NAs, their “hot spots” numbers will be 2N, 6N–3 and 7N–5, respectively. The “hot spots” numbers of the 111, 212 and 222 NAs will linearly increase with increased Au NPs numbers (Fig. S8). Moreover, the proportion of the “hot spots” numbers among 111, 212 and 222 NAs will approach to 2:6:7 when the numbers of the assembled Au NPs were increased to some degree, which is nearly in agreement with the experimental results (18:49:69) in Fig. 5. The “hot spots”–number–depended SERS properties of the Au NAs will be available for SERS quantitative analysis. The accurate SERS intensity of the Au NAs can be therefore predicted only through designing specific assembly mode and calculating its “hot spots” numbers.

## Conclusions

In summary, we have demonstrated that a simple, effective and general DNA–directed self–assembly strategy can produce well–organized and geometrically controlled Au NAs, in which internal–modified dithiol ssDNA Au NPs and molecule quantity of thiol–modified ssDNA grafted to Au NPs play important roles. Through selecting available Au–DNA self–assembly units, geometrical structures of the Au NAs can be well tailored from 1D to quasi–2D and 2D. Au–B–A conjugates give 1D and quasi–2D Au NAs while 2D Au NAs can be obtained by A–B–Au–B–A building blocks. Absorption spectra indicate that the Au NAs have different surface plasmon resonance properties from isolated Au NPs and their intensities are associated with their geometrical structures.

The SERS measurements and 3D–FDTD calculation results reveal that the geometrically controllable Au NAs have regular and linearly dependent SERS properties. Besides the fixed size (ca. 14.4 nm) and interparticle distance (ca. 2.0 nm), the SERS intensities acquired from Au NAs only depend on the “hot spot” numbers of different assembly structures. The “hot spots”–number–depended SERS properties of Au NAs open a new avenue for quantitative analysis of SERS intensity. The self–assembly strategy described here holds promise for constructing an ideal SERS substrate of high sensitivity, stability, reproducibility and independent light polarization through using larger sized Au NPs (60–100 nm) to construct well–defined, ordered, periodical, smaller interparticle distance (around 1.0 nm) and large–sized 2D Au (micrometer scale) NAs.

## Methods

### Materials

1×TBE buffer: tris(hydroxymethyl)aminomethane (Tris, 89 mM), boric acid (89 mM) and ethylenediaminetetraacetic acid (EDTA, 2 mM), pH 8.0. Chlorauric acid (HAuCl_4_), bis(p–sulfonateophenyl)phenylphosphine dihydrate dipotassium (BSPP), Rhodamine 6G (R6G), Tris and dithiothretol (DTT) were obtained from Sigma–Aldrich (St Louis, MO, USA). EDTA, sodium citrate acid (Na_3_C_6_H_5_O_7_), boric acid, sodium chloride (NaCl), hydrochloric acid (HCl), concentrated sulphuric acid (H_2_SO_4_) and methanol were analytical grade reagent from Guangdong Guanghua Chemical Reagent Co. (China). DNA oligonucleotides were synthesized by Sangon Biotech and the sequences were listed in Table S1. All the reagents were used as received without further purification. Milli–Q water (>18.0 MΩ cm) was used to prepare all aqueous solutions.

### Synthesis of BSPP–capped Au NPs

Au NPs with an average diameter of ca. 14.4 nm were prepared according to the literatures with modifications^46^. Briefly, 50 mL of 0.25 mM HAuCl_4_ was first heated to boiling and kept stirring, followed by the quick addition of 1.5 mL of 40 mM Na_3_C_6_H_5_O_7_. Then, the mixture turned ruby red after 30 min, indicating the formation of Au NPs. 50 mL of the cooled Au NPs solution was next incubated with 6 mg BSPP for 12 h under stirring. Subsequently, the BSPP–capped Au NPs were concentrated with 0.5 g NaCl and centrifuged at 10000 rpm for 15 min. Finally, the BSPP–capped Au NPs were resuspended in 0.5 mM BSPP, the final concentration of which was 0.1 μM.

### Synthesis of the Au NPs–DNA conjugates

The target thiolated DNA sequences (i.e., B–A, A´ and B´) were lengthened with an excess of their corresponding DNA strands in 40 mM NaCl solution, respectively. That is to say, B–A was lengthened with EXT–B(1), B(2), B(3), B(4), B(5) and B(6); A´ was lengthened with EXT–A´(1), B(3), B(4), B(5), B(6) and A´(6); B´ was lengthened with EXT–B´(1), B(2), B(3), B(4), B(5) and B(6). Of special emphasis here, the concentration ratio of thiol-modified DNA strand (i.e., B-A, A´ and B´) and the first lengthening DNA strand is 1:3, and the following lengthening strands were added with 1.5 times increase in concentration. Then, six lengthening strands and thiol-modified DNA were mixed and heated at 85 °C for 10 min and incubated for 3 h at room temperature. Next, the BSPP–capped Au NPs and as–prepared lengthened B–A, A´ and B´ were mixed with the concentration ratio of 1:3 and were subsequently incubated for 12 h at room temperature after mixing 1 mM BSPP and 30 mM NaCl. These incubated solutions were loaded in 3% agarose gels (1×TBE as running buffer) after adding one volume of glycerol for five volumes of loaded sample. The gels were run at 80 V for 70 min before obtaining isolated bands. Finally, the Au–DNA conjugates (Au–A´, A´–Au–A´, Au–B´, B´–Au–B´, Au–B–A and A–B–Au–B–A) were prepared by cutting these different bands and extracting with 1×TBE in 4 °C, respectively.

**Synthesis of different Au NPs assemblies.** The Au–A´, A´–Au–A´, Au–B´, B´–Au–B´, Au–B–A and A–B–Au–B–A concentrations were first quantified by UV–visible absorption spectroscopy. Then, Au–A´, Au–B–A and Au–B´ solutions with the same concentrations were mixed in 20 mM NaCl. Finally, 111 NAs were obtained by heating the mixture at 55 °C for 10 min and incubating for 12 h at room temperature. Similarly, 121, 212 and 222 NAs were acquired through mixing Au–A´, A–B–Au–B–A and Au–B´; A´–Au–A´, Au–B–A and B´–Au–B´; A´–Au–A´, A–B–Au–B–A and B´–Au–B´, respectively.

### Characterization

TEM was carried out with a Hitachi H–7500 microscope operated at 80 kV. UV-vis absorption spectra were performed using on a Hewlett–Packard 8452 diode array spectrometer (U–3010). XPS spectra were obtained by an ESCALAB 250 X-ray photoelectron spectrometer (Axis Ultra DLD, UK). Particle size distribution was recorded by Malvern Nano-ZS Laser Particle Size Analyzer (UK). SERS spectra were acquired using a confocal microprobe Raman system (LabRAM Aramis, France) operated with a He–Ne laser (632.8 nm) and the data acquisition times were 10 and 20 s for the Au NAs and isolated NPs, respectively. The SERS samples were prepared by adding 3.8 μL of 10^–4^ M R6G into 15 μL of 4.9 × 10^–9^ M isolated NPs and 111, 212 and 222 Au NAs, respectively, and then the mixed solutions were dropped and dried on glass for SERS measurements.

## Additional Information

**How to cite this article**: Yan, Y. *et al.* Internal-Modified Dithiol DNA-Directed Au Nanoassemblies: Geometrically Controlled Self–Assembly and Quantitative Surface–Enhanced Raman Scattering Properties. *Sci. Rep.* **5**, 16715; doi: 10.1038/srep16715 (2015).

## Supplementary Material

Supplementary Information
