# Supplementary material for: Internal-Modified Dithiol DNA–Directed Au Nanoassemblies: Geometrically Controlled Self–Assembly and Quantitative Surface–Enhanced Raman Scattering Properties
Source: Sci Rep. 2015 Nov 19;5:16715. doi: 10.1038/srep16715 (PMC4652228; doi:10.1038/srep16715)
Supplement: Supplementary Information [file srep16715-s1.doc]

**Supporting Information**

**Internal-Modified Dithiol DNA–Directed Au Nanoassemblies: Geometrically Controlled Self–Assembly and Quantitative Surface–Enhanced Raman Scattering Properties**

*Yuan Yan,^1,‡^ Hangyong Shan^2,‡^, Min Li^1,‡^, Shu Chen^2^, Jianyu Liu,^1^,* Yanfang Cheng,^1^ Cui Ye, Zhilin Yang,^2^,* Xuandi Lai^1^ & Jianqiang Hu^1^,**

^1^ Department of Chemistry, College of Chemistry and Chemical Engineering, South China University of Technology, Guangzhou, 510640, China. E-mail: [jqhusc@scut.edu.cn](mailto:jqhusc@scut.edu.cn); [jyliu@scut.edu.cn](mailto:jyliu@scut.edu.cn).

^2^ Department of Physics, Xiamen University, Xiamen 361005, China. E-mail: [zlyang@xmu.edu.cn](mailto:zlyang@xmu.edu.cn) .

^‡^ Yuan Yan, Hangyong Shan and Min Li equally contributed to this work.

**Table S1 |** DNA sequences for the preparation of geometrically controlled Au nanoassemblies. The medium disulfide (-S-S-) group in the B-A DNA sequence was coupled with alkyl chains, marked with red in Figure S1.


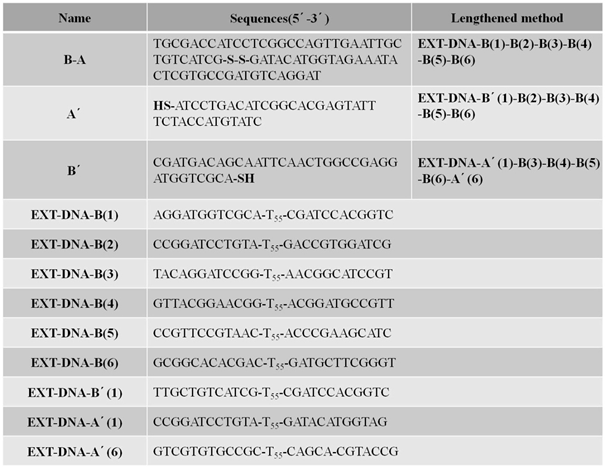


**Table S2 |** FDTD calculated polarization and electric-field enhancement results of 111, 212 and 222 NAs prepared by different assembly modes.

| Au NAs | X-polarization maxmum electric-field  (V/m) | X-polarization electric-field enhancement factor  (|E_loc_/E_0_|^4^) | Y- polarization maxmum electric-field  (V/m) | Y- polarization electric-field enhancement factor (|E_loc_/E_0_|^4^) |
| --- | --- | --- | --- | --- |
| **111** | 17.4 | 9.2 × 10^4^ | 5.2 | 7.3 × 10^2^ |
| **212** | 17.5 | 9.4 × 10^4^ | 10.2 | 1.1 × 10^4^ |
| **222** | 14.1 | 4.0 × 10^4^ | 12.3 | 2.3 × 10^4^ |

**Figure S1**


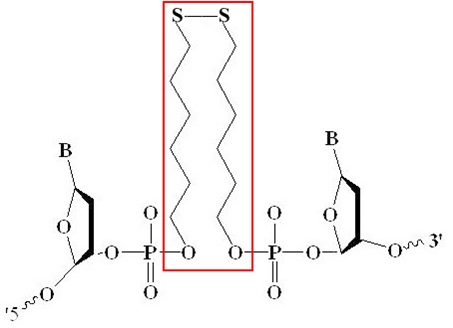


**Figure S1 |** Molecular structure of a DNA sequence with medium disulfide (-S-S-) group.

**Figure S2**


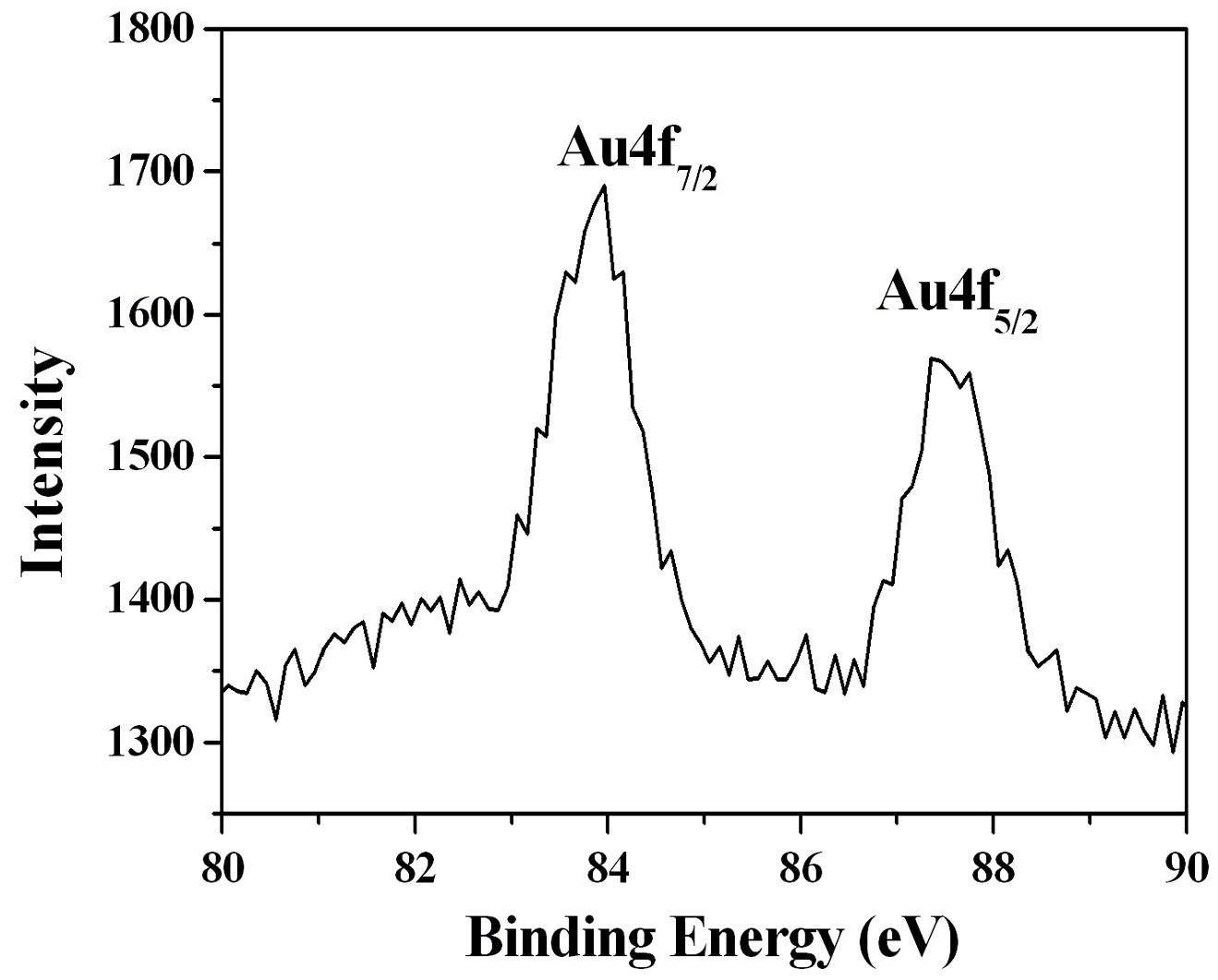


**Figure S2 |** XPS spectra of the Au lines obtained in powder Au nanoparticles synthesized by the present method.

**Figure S3**


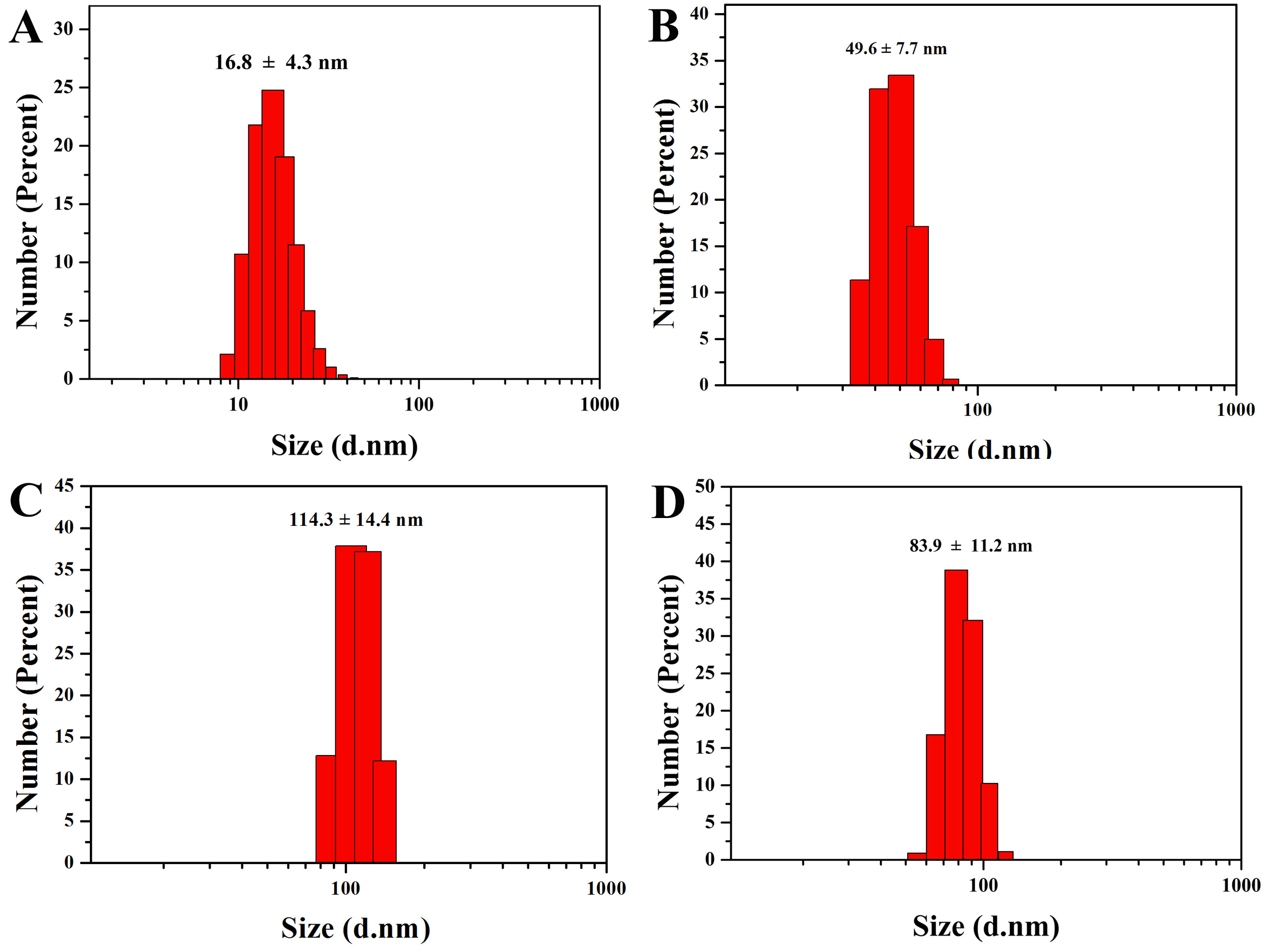


**Figure S3 |** DLS spectra of (A) Au NPs and (B-D) 111, 212 and 222 Au NAs solutions.

**Figure S4**


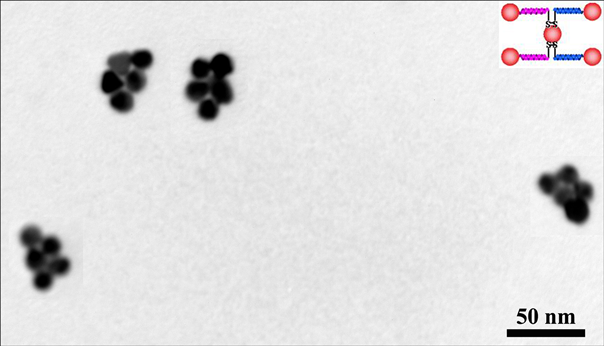
**Figure S4 |** TEM image of tetrahedral structured 121 Au NAs prepared with Au-**B´**, **B-A**-Au-**B-A** and Au-**A´** conjugates (concentration ratio: 2:1:2). Inset: schematic illustration of tetrahedral structured 121 Au NAs constructed with Au-**B´**, **B-A**-Au-**B-A** and Au-**A´** conjugates.

**Figure S5**


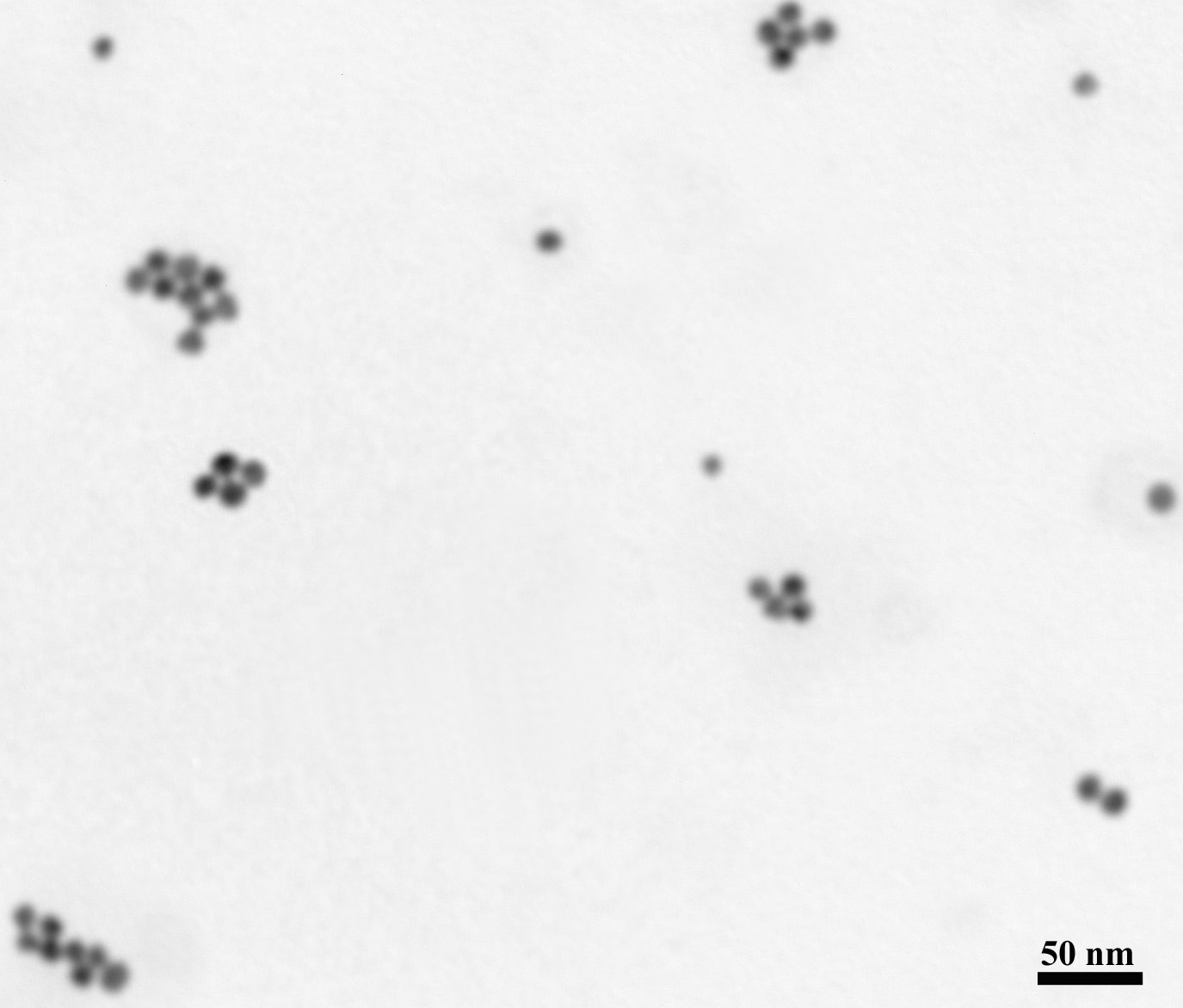


**Figure S5 |** TEM image of 222 Au NAs constructed using the 1:1.5:1 concentration ratio of A´–Au–A´, A–B–Au–B–A and B´–Au–B´ building blocks.

**Figure S6**


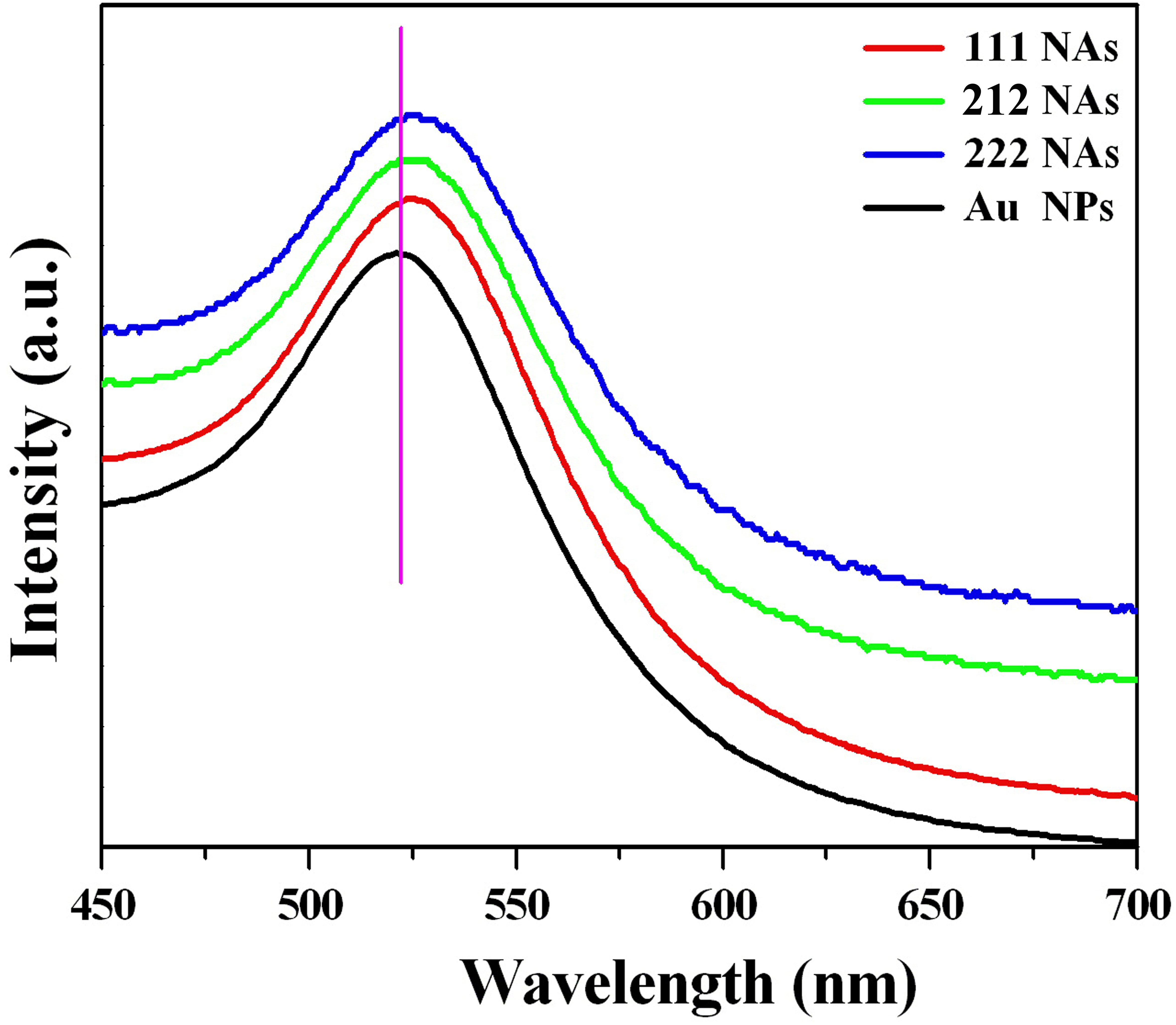


**Figure S6 |** UV-vis absorption spectra of the Au NPs and **111**, **212** and **222** NAs solutions prepared by the present methods, respectively.

**Figure S7**


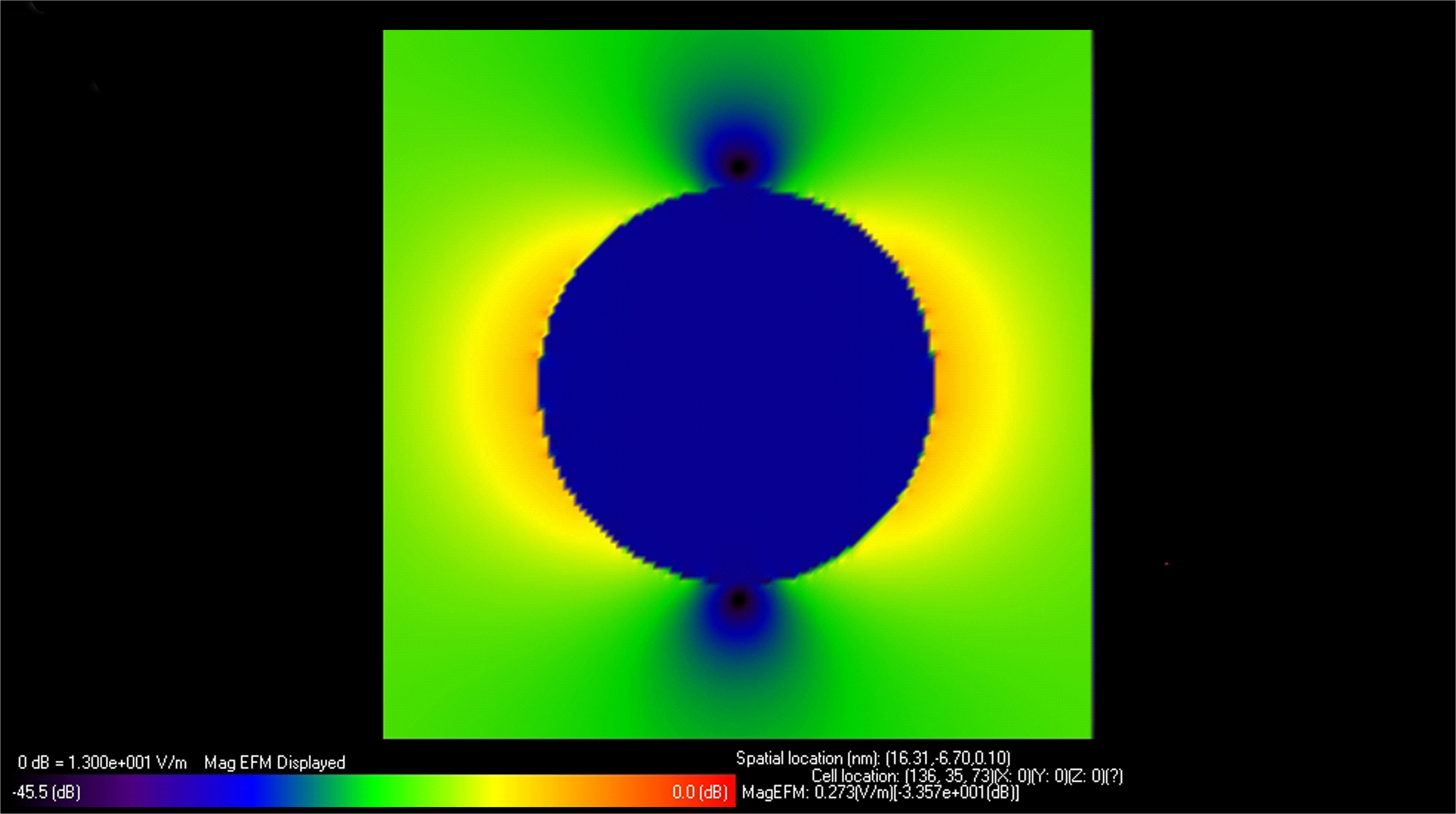


**Figure S7 |** 3D-FDTD simulated electric field patterns of the isolated Au NPs prepared by the present method.

**Figure S8**


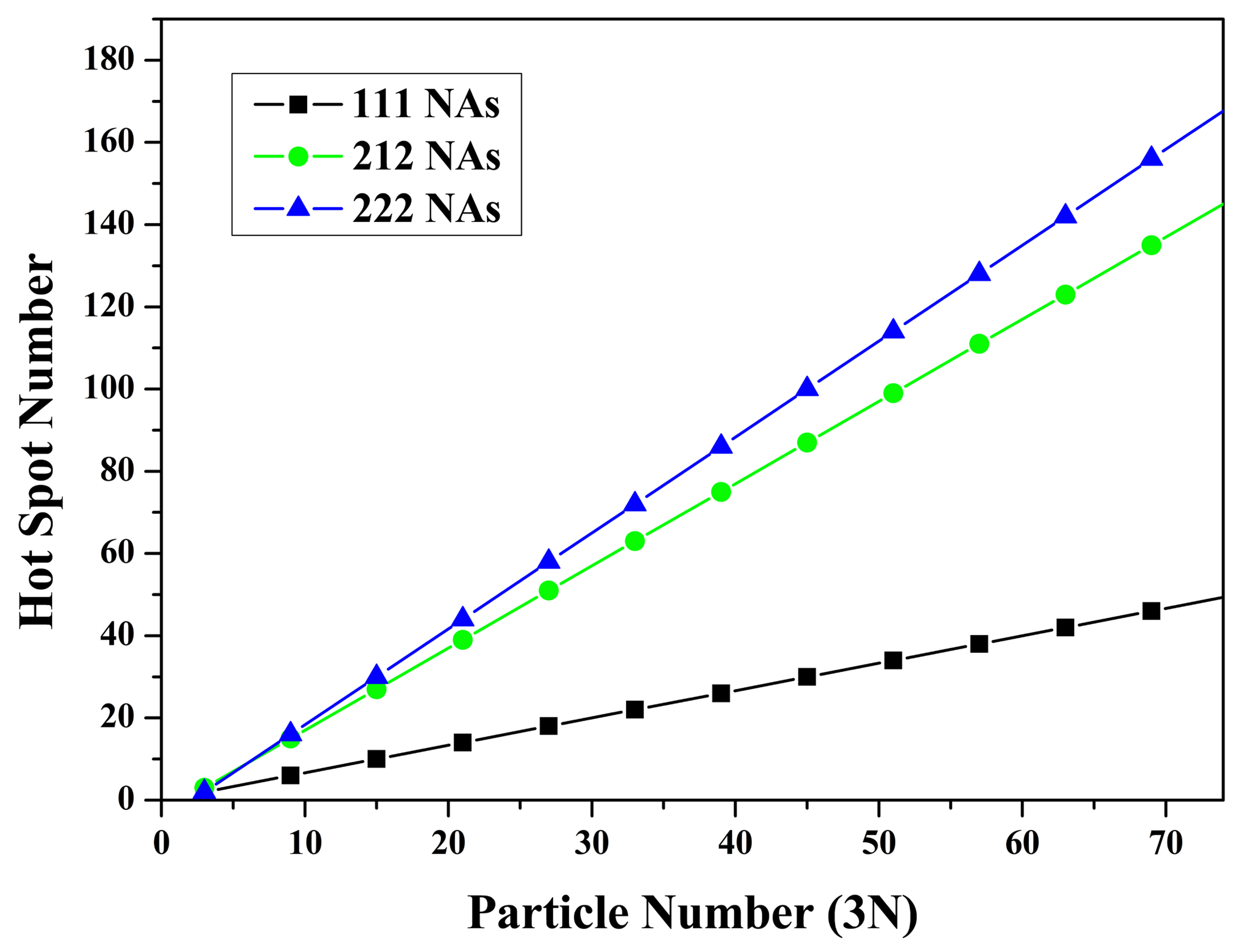


**Figure S8 |** Plots of hot spot numbers of Au NAs obtained by different assembling methods versus assembled particle numbers.
